# Improvement of mitochondrial function mediated the neuroprotective effect of 5-(4-hydroxy-3-dimethoxybenzylidene)-2-thioxo-4-thiazolidinone in rats with cerebral ischemia-reperfusion injuries

**DOI:** 10.18632/oncotarget.18048

**Published:** 2017-05-22

**Authors:** Mingyang Wang, Lu Feng, Ji Zheng, Junya Liu, Shujie Fan, Jun Zhao, Nan Yang, Yanyong Liu, Zhanjun Yang, Caiying Ye, Pingping Zuo

**Affiliations:** ^1^ Department of Pharmacology, Institute of Basic Medical Sciences, Chinese Academy of Medical Sciences & Peking Union Medical College, Beijing 100005, China; ^2^ Department of Human Anatomy, Research Laboratory of Human Anatomy, Baotou Medical College, Inner Mongolia, Baotou 014040, China

**Keywords:** 5-(4-hydroxy-3-dimethoxybenzylidene)-2-thioxo-4-thiazolidinone, neuroprotection, cerebral ischemia reperfusion, mitochondrial function, synaptic activity

## Abstract

Deficits in mitochondrial function is a critical inducement in the major pathways that drive neuronal cell death in ischemic process particularly. Drugs target to improve the mitochondrial function may be a feasible therapeutic choice in treatment with ischemic diseases. In the present study, we investigated whether 5-(4-hydroxy-3-dimethoxybenzylidene)-2-thioxo-4-thiazolidinone (RD-1), a compound derived from rhodanine, could protect against ischemic neuronal damage via improving mitochondrial function. We tested the neuroprotective effect of RD-1 both in rats modeled by middle cerebral artery occlusion reperfusion *in vivo* and in primary cortical neurons subjected to hypoxia/reperfusion injury *in vitro*. Results showed that treatment with RD-1 for 14 days remarkably reduced infarct size, decreased neurological deficit score and accelerated the recovery of somatosensory function *in vivo*. Meanwhile, RD-1 also increased the cellular viability after 48 h treatment *in vitro*. In addition, RD-1 protected the primary cortical neurons against mitochondrial damage as evidenced by stabilizing the mitochondrial membrane potential and reducing the overproduction of reactive oxygen species. Furthermore, hypoxia/reperfusion injury induced damaged mitochondrial axonal transport and consequently neurotransmitter release disorder, which were ameliorated by RD-1 treatment. Besides, RD-1 inhibited the downregulation of proteins related with mitochondrial transport and neurotransmitter release induced by ischemic injury both *in vivo* and *in vitro*. The obtained data demonstrated the neuroprotective effect of RD-1 and the involved mechanisms were partially attributed to the improvement in mitochondrial function and the synaptic activity. Our study indicated that RD-1 may be a potential therapeutic drug for the ischemic stroke therapy.

## INTRODUCTION

Ischemic stroke is one of the leading causes of disability and mortality in the world, which exerts a profoundly negative impact on both patients and society [1, 2]. Thrombolysis with tissue plasminogen activator is the only effective therapeutic drug approved by the European Medicines Agency and the US Food and Drug Administration for the treatment of ischemic stroke [3]. However, the short time window for patients limits its widespread application so that only approximately 5% of patients benefit from it [4–6]. Therefore, it is urgent to develop safe and effective drugs with an extended therapeutic window for stroke therapy.

Ischemia is a complicated process that ultimately leads to neuronal cell death via multiple mechanisms. Therein, mitochondria dysfunction is crucial to driving neuronal cell death, including impairment of the ability to generate ATP, the probable induction of the mitochondrial permeability transition during early reperfusion and the release of pre-apoptotic factors [7, 8]. With that comes the loss of the mitochondrial membrane potential (MMP) and the excessive production of radical oxidative species (ROS) [9]. The later feature may even exacerbate the damage and make cells trap in a vicious cycle. Thus, protecting mitochondria from cerebral ischemic injury and enhancing its capability of responding to injury has great meanings.

We previously synthesized and screened a derivative of rhodanine via chemical modifications, named 5-(4-hydroxy-3-dimethoxybenzylidene)-2-thioxo-4-thiazolidinone (RD-1). Further study showed that RD-1 possessed protective effects on movement disorder and mitochondrial dysfunction in Parkinson's disease mice model. The compound also promoted neuronal functional recovery via attenuating impaired mitochondrial transport and vesicle release dysfunction evoked by MPP^+^ cytotoxicity in cultured primary mesencephalic neurons [10].

Considering the similarities in the pathologic mechanisms underlying both cerebral ischemia and Parkinson's disease, we investigate the potential therapeutic effect of RD-1 on focal cerebral ischemia reperfusion injury both in rats with middle cerebral artery occlusion (MCAO) model *in vivo* and in primary cortical neurons (PCNs) with oxygen-glucose deprivation and reoxygenation (OGD/R) injury *in vitro*. We also further revealed the underlying mechanisms whereby RD-1 alleviated mitochondrial damage caused by cerebral ischemia reperfusion injury.

## RESULTS

### RD-1 reduced ischemic injury and improved functional recovery after MCAO in rats

**Figure 1:**
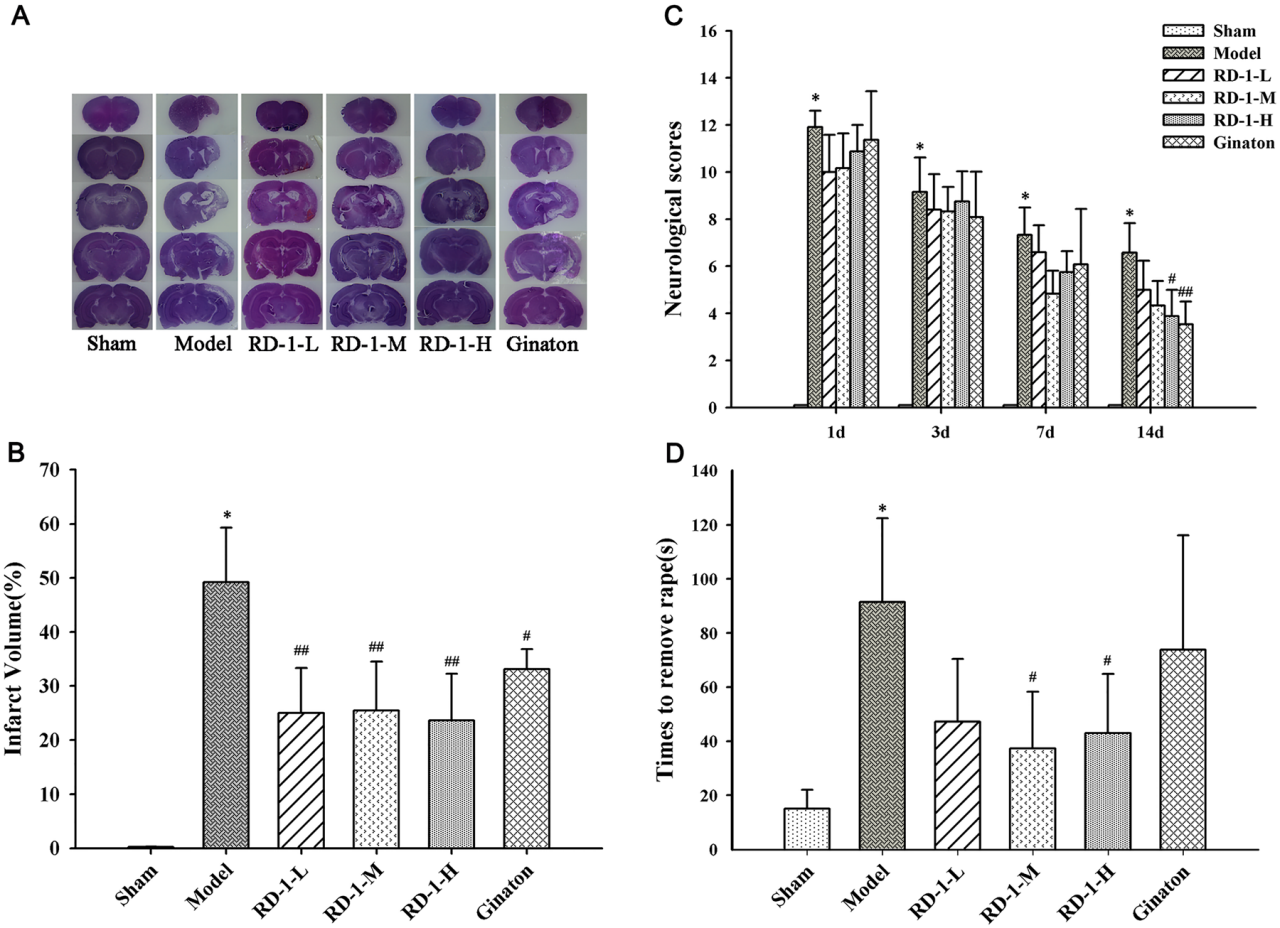
Protective effect of RD-1 against brain injury after cerebral ischemia reperfusion in MCAO rats The NSS scores were tested at 1, 3, 7 and 14 days after reperfusion. Adhesive-removal test was evaluated at 14 days after reperfusion. **(A)** Representative infarct volume measured by HE staining at 14 days after reperfusion. **(B)** Quantitative evaluation of infract volume in experimental groups. **(C)** NSS scores. **(D)** Adhesive-removal for somatosensory test. Data were expressed as mean ± SD, *P < 0.001 vs. Sham; ^#^*P* < 0.05, ^##^*P* < 0.01 vs. Model; n = 8-10.

Firstly, we explored the neuroprotective effect of RD-1 in rats with MCAO reperfusion injury. 14 days after reperfusion, there was no observed cerebral injury in the sham group, whereas the model group showed a significant area of infarct (49%). Post-treatment with RD-1 significantly decreased infarct volumes to 25%, 25% and 24% at 5 mg/kg, 10 mg/kg and 20 mg/kg dose, respectively, as the positive control Ginaton did (Figure 1A, 1B). Meanwhile, by employing a variety of tests including the Neurological Severity Scores (NSS) and adhesive-removal tests, we also found that RD-1 improved neurological functional performance after MCAO. Results showed that the rats in model group exhibited the highest neurological deficit score whereas rats administered with 20 mg/kg of RD-1 significantly reduced the scores by half (Figure 1C). Rats exposed to 10 mg/kg and 20 mg/kg of RD-1 also expended shorter times (37.27 s and 42.98 s) to remove stimulus compared with the model group (91.45 s) (Figure 1D). Therefore, RD-1 treatment protected the brain against MCAO reperfusion injury.

### RD-1 ameliorated PCNs death *in vitro* after OGD/R injury

**Figure 2:**
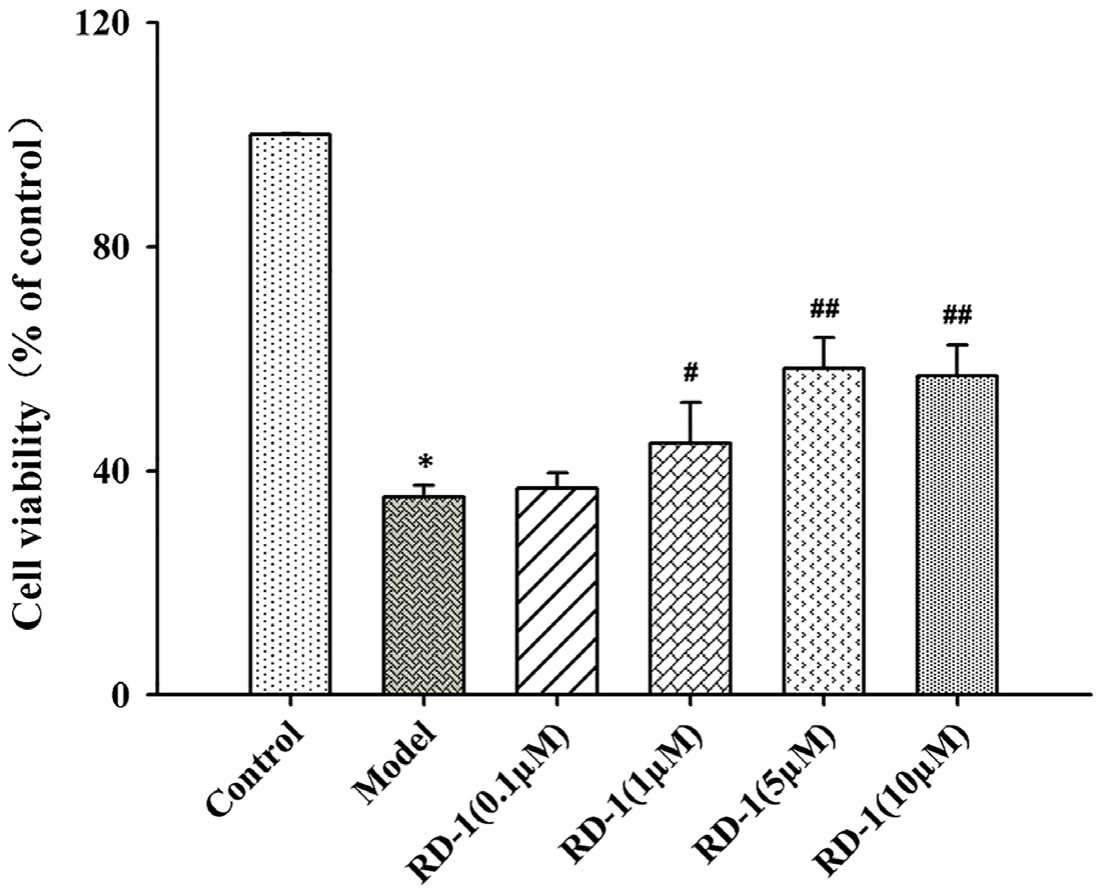
Protective effect of RD-1 against hypoxia-induced damage in PCNs subjected to OGD/R injury Cells were post-treated with serial concentrations of RD-1 (0, 0.1, 1, 5 and 10 μmol/L). Cell viability was measured by CCK-8 assay after 48 h treatment. The values were represented as mean ± SD, *P < 0.001 vs. Control; ^#^*P* < 0.05, ^##^*P* < 0.001 vs. Model; n = 3.

To verify the neuroprotective effect of RD-1, OGD/R model was applied to mimic the ischemia-reperfusion injury on PCNs followed by CCK-8 cell viability assay. Cell survival was suppressed when PCNs were subjected to OGD/R injury. However, treatment with different concentrations of RD-1 (1, 5 and 10 μmol/L) for 48 h exerted a protective effect against OGD/R induced damage, which recovered the cell viability to 44%, 58% and 57%, respectively (Figure 2).

### RD-1 reduced intracellular ROS generation and stabilized MMP in PCNs subjected to OGD/R

**Figure 3:**
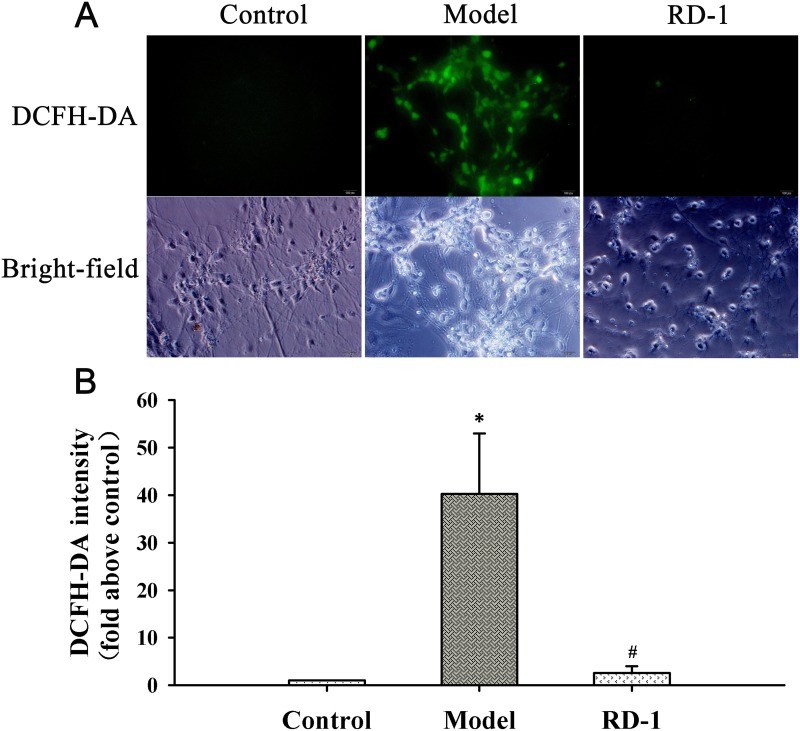
RD-1 reduced the ROS generation in PCNs subjected to OGD/R injury ROS generation was measured by fluorescent probe DCFH-DA after 12 h treatment in the presence or absence of 5 μmol/L RD-1. The concentration of RD-1 was selected based on the cell viability analysis. **(A)** Representative image of ROS fluorescence in each group (magnification 40×, Scale bar = 100 μm). **(B)** Quantitative analysis of relative DCFH-DA fluorescence intensity. The values were represented as mean ± SD, ^*^P < 0.001 vs. Control; ^#^*P* < 0.001 vs. Model, n = 3.

Both ROS overproduction and MMP decline are important early markers for mitochondrial dysfunction. To investigate the influence of RD-1 on the mitochondrial damage, we detected ROS generation intracellular and the fluctuation of MMP. After 12 h reperfusion, OGD/R induced a dramatic rise in ROS as shown in Figure 3. However, DCF fluorescence was barely detectable in RD-1 treated cells, indicating that RD-1 attenuated OGD/R induced ROS production in PCNs.

**Figure 4:**
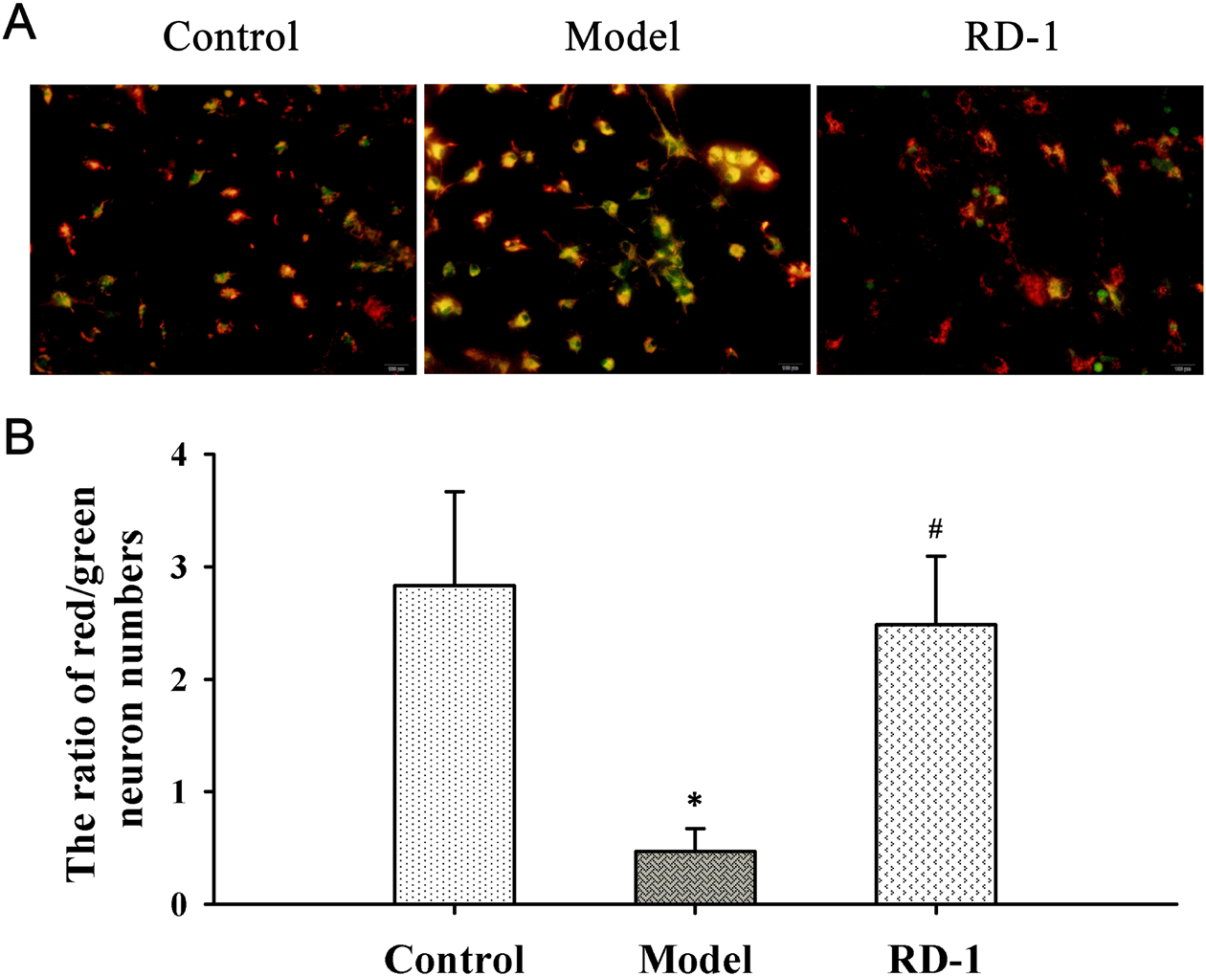
RD-1 stabilized MMP in PCNs subjected to OGD/R injury MMP was measured by JC-1, an indicator of mitochondrial function. Red fluorescence represents the mitochondrial aggregate JC-1 and green fluorescence indicates the monomeric JC-1. The concentration of RD-1 was selected based on the cell viability analysis. **(A)** Representative image of JC-1 labeled MMP staining in each group (magnification 40×, Scale bar = 100 μm). **(B)** Quantitative analysis of MMP. The values were represented as mean ± SD, *P < 0.001 vs. Control; ^#^*P* < 0.001 vs. Model, n = 3.

We investigated the effect of RD-1 upon MMP by using cationic dye JC-1. As shown in Figure 4A, cells with intact MMP appeared fluoresce red. In 12 h after reperfusion, OGD/R treated cells of the model group showed high proportion of green fluorescence neurons as JC-1 turns to monomeric form in injured or dead cells. It is noted that cells appeared red following RD-1 treatment and the ratio of red/green neuron numbers was markedly elevated from 0.47 to 2.48 (Figure 4B), suggesting that RD-1 treatment stabilized the fluctuation of MMP evoked by OGD/R injury.

### RD-1 improved the impaired axonal mitochondrial transport

**Figure 5:**
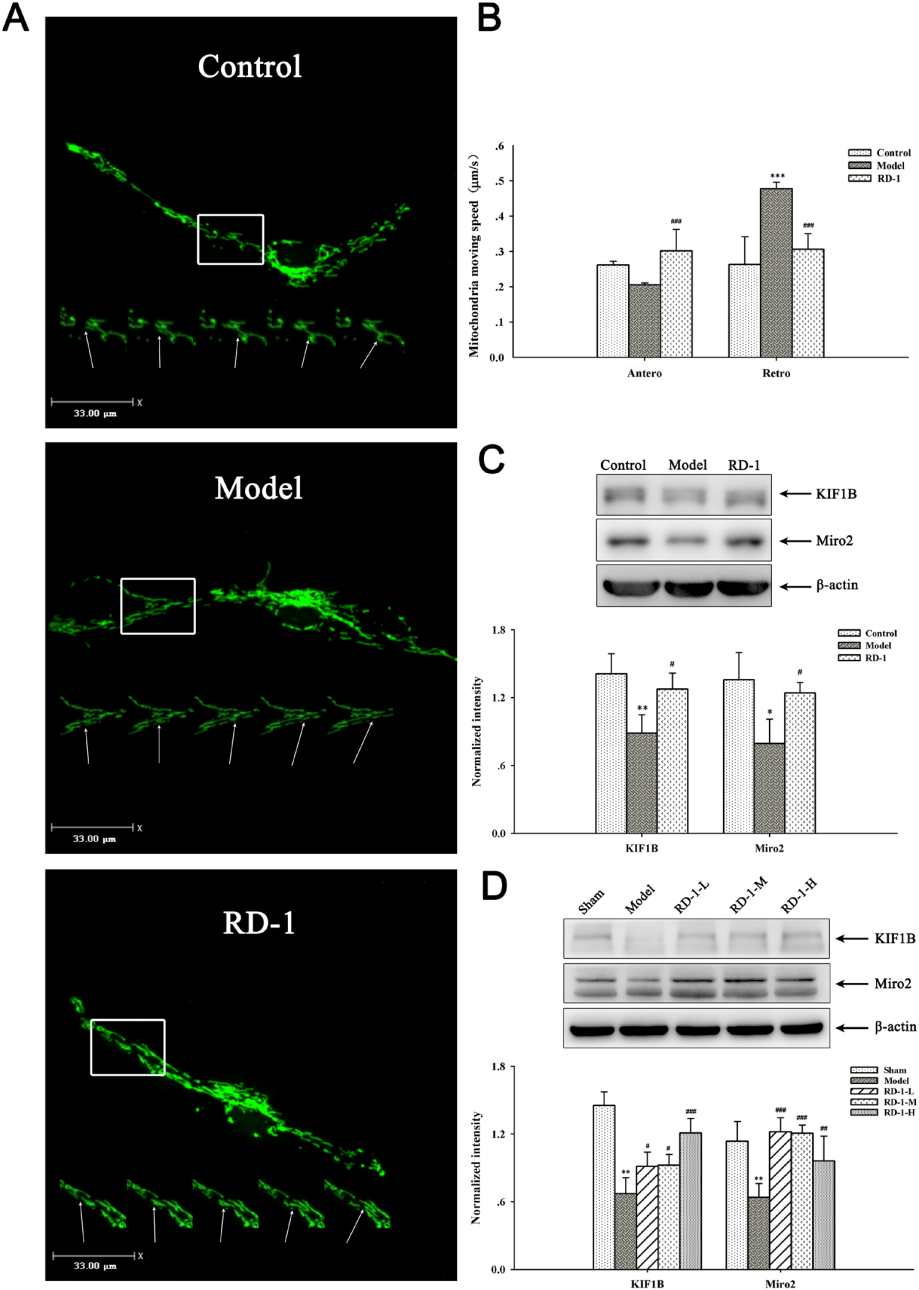
Effects of RD-1 on the impaired axonal mitochondrial transport **(A)** Representative view frame of mitochondrial movement along the axon (Scale bar = 33 μm), mitochondria move along the axon shown in a rectangle area; the arrow pointed to the track of an retrograde moving mitochondrion. **(B)** Mean speed of motile mitochondria in each group. **(C) and (D)** Expression of KIF1B and Miro2 in PCNs and ischemic cortex, the intensity of each band was normalized to that of β-actin. The values were represented as mean ± SD, *P < 0.05, **P < 0.01 vs Control or Sham; ^#^*P* < 0.05, ^##^*P* < 0.01 vs Model, n = 3.

It is well-known that energy metabolism is the first to be affected during the OGD/R injury. While mitochondria are the major site of ATP production for cell survival and many other vital cellular functions, regulating of mitochondrial transport is essential to meet the altered metabolic requirements and to remove aged and damaged mitochondria or replenish healthy ones to distal terminals [11]. Hence we explored the effects of RD-1 on the mitochondrial axonal transport in PCNs. As shown in Figure 5A and 5B, the speed of anterograde transport of mitochondria was declined to 0.20 μm/s, whereas retrograde transport of mitochondria was remarkably elevated to 0.48 μm/s at 12 h after OGD/R injury. By contrast, this damage was reversed dramatically by 5 μmol/L RD-1 treatment, which significantly improved the mitochondrial anterograde transport (0.30 μm/s) and slowed down the retrograde transport (0.30 μm/s).

To get an insight of the molecular involvement in the improvement effect of RD-1 on the damaged mitochondrial transport, we detected the expression of KIF1B and Miro2 proteins in PCNs and ischemic cortex, which act as motor and receptor in complexes that driving the mitochondrial anterograde movements, respectively [12, 13]. Results showed that OGD/R injury remarkably decreased KIF1B and Miro2 level, but RD-1 treatment alleviated these pathological changes (Figure 5C). Similar data were obtained in *in vivo* experiments (Figure 5D).

### RD-1 promoted the synaptic activity

**Figure 6:**
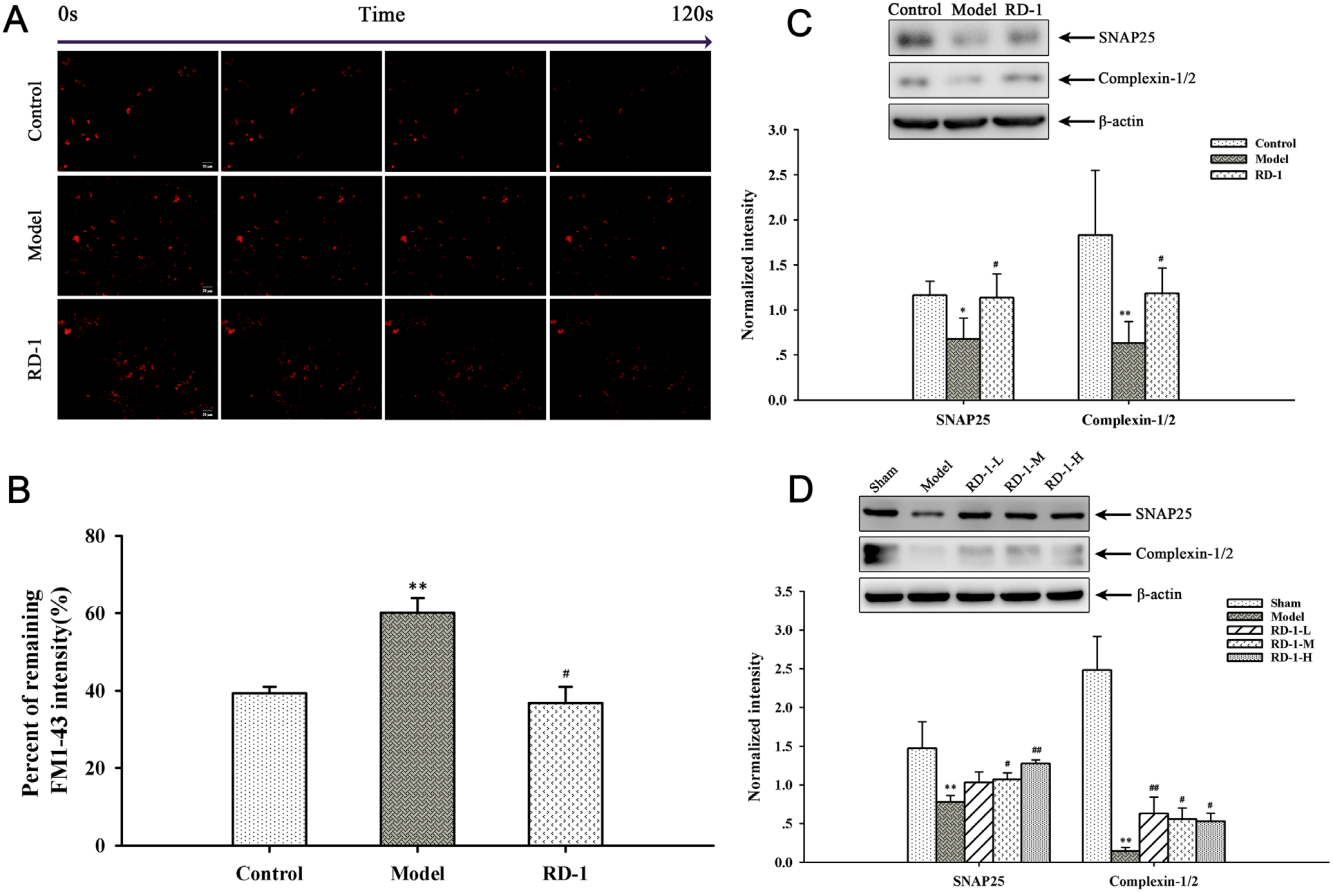
Effects of RD-1 on the synaptic vesicle release FM1-43 dye was used to image synaptic vesicle release. **(A)** Representative time-relapse destaining of synaptic boutons of different groups (magnification 20×, Scale bar = 50 μm). **(B)** Analytical results of synaptic vesicle release efficacy as indicated by percentage of remaining fluorescence intensity. **(C)** and **(D)** Expression of SNAP25 and Complexin-1/2 in PCNs and ischemic cortex, the intensity of each band was normalized to that of β-actin. The values were represented as mean ± SD, *P < 0.05, **P < 0.01, ***P < 0.001 vs Control or Sham; ^#^*P* < 0.05, ^##^*P* < 0.01, ^###^*P* < 0.001 vs Model, n = 3.

The condition of synaptic activity makes a great contribution to the normal physiological function in neurons. Mitochondrial transport in axons and distribution at synapses is closely associated with synaptic activity. Abnormal removal of mitochondria from axon terminals could result in aberrant synaptic transmission [14, 15]. To further reveal the secondary effect of mitochondrial damage observed in above mentioned experiments, FM1-43 was employed to directly measure synaptic vesicle release in PCNs. Here we found that OGD/R injury significantly reduced the efficacy of neurotransmission. As more than 60% neurotransmitter of model cells were not released in 2 min, while the value in RD-1-exposure cells was only 37%, which was closed to control cells (Figure 6A and 6B). The result confirmed that RD-1 treatment markedly elevated the efficacy of neurotransmission and improved neuronal terminal activities, suggesting additional evidence for its neuroprotective effects in OGD/R injury.

Concurrently, the efficacy of neurotransmitter release is bound up with the expression of proteins associated with synapse, such as SNAP25 and Complexin-1/2. SNAP25 is one of the core proteins forming the SNARE complex and Complexin-1/2 is SNARE complex-binding protein, both of which are involved in the exocytotic release of neurotransmitters during synaptic transmission [16, 17]. The expression of SNAP25 and Complexin-1/2 were down-regulated by OGD/R while significantly elevated by RD-1-exposure (Figure 6C). The western blot results of our *in vivo* experiments were in accordance with the *in vitro* study, as shown in Figure 6D.

## DISCUSSION

The present study evaluated the potential protective effects of RD-1 on the cerebral ischemia/reperfusion injuries both *in vivo* and *in vitro*. Like the positive control Ginaton, RD-1 was found to protect the brain from ischemic injury as evidenced by reducing infarct size, improving neurological functions, and accelerating the recovery of somatosensory function in MCAO reperfusion rats. Meanwhile, the neuroprotective effect of RD-1 was verified in rat PCNs subjected to OGD/R *in vitro* by increasing neuron cellular viability after ischemia.

To clarify the underlying mechanism, we firstly focused to explore the improvement effect of RD-1 on the mitochondrial function. Many mechanisms involved in ischemic cell death are associated with mitochondrial dysfunction [18]. In ischemic and post-ischemic brain, mitochondria are among the first ones to suffer the damage, followed by numerous ROS outburst and collapse of the MMP [19]. Since mitochondria are both generators and targets of ROS in cells, all of these damages exacerbate the ischemic injury and make neurons trap in a vicious cycle [20]. In our study, we found amount of ROS outburst intracellular and MMP declined significantly in PCNs after OGD/R injury. However, both impairments were mitigated by 5 μmol/L RD-1 treatment after 12 h, which reduced the generation of ROS and stabilized the MMP.

As the “power house” of the cell, mitochondria must methodically transport to distal regions with high-energy requirements in neurons to maintain cellular homeostasis and survival [21]. Meanwhile, impaired mitochondria, as identified by a fall in MMP, may need to be removed from distal axons [22]. Therefore, regulation of mitochondrial transport is essential to meet altered metabolic requirements and to remove aged and damaged mitochondria or replenish healthy ones to distal terminals [11]. At 12 h after OGD/R injury, we noticed that the mitochondrial anterograde transport in PCNs was inhibited and the retrograde transport was enhanced on the contrary. RD-1 significantly reversed these changes. We also detected the expression of KIF1B (motor) and Miro2 (receptor), which compose the motor/adaptor/receptor complexes to ensure targeted mitochondrial trafficking and precise regulation of their distribution in response to changes in neuronal activity [23]. Both *in vivo* and *in vitro* results showed that ischemic injury decreased KIF1B and Miro2 protein levels, indicating weakened mitochondrial anterograde movements from another angle. While this effect was mitigated by RD-1 treatment.

Mitochondrial transport deficit results in the loss of mitochondria from synaptic terminals, where mitochondria help to maintain neurotransmission by producing ATP and buffering Ca^2+^ at synapses [24–26]. Since presynaptic and postsynaptic sites are subcellular regions with high energy demands, a lack of energy is bound to the disorder of synaptic activity [27]. We then further investigated the effect of RD-1 on synaptic vesicle release, which reflects neuronal functional output. Data showed that RD-1 remarkably relieved the impairment in vesicle release efficacy induced by OGD/R injury in PCNs. Our *in vitro* and *in vivo* western blot results illustrated that OGD/R and MCAO injury also down-regulated the expression of SNAP25 and Complexin-1/2, suggesting that neurotransmitter release in ischemic PCNs and cortex may be damaged. RD-1 treatment could increase the expression of both proteins, repair the injury.

In summary, the present study firstly demonstrated the neuroprotective effects of RD-1 on cerebral ischemia/reperfusion induced brain injury *in vivo* and *in vitro*. And the effect at least partially due to the improvement ability of attenuating mitochondrial dysfunctions and promoting the synaptic activity. Our research showed that RD-1 may be a candidate drug for cerebral stroke therapy, however other potential involved signaling pathways should be illustrated in the following research.

## MATERIALS AND METHODS

### Animals

The animal study was conducted in accordance with the guidelines established by the National Institutes of Health for the care and use of laboratory animals and approved by the Animal Care Committee of the Peking Union Medical College and Chinese Academy of Medical Sciences. Adult male Sprague-Dawley rats weighing 250-270 g were purchased from Academy of Military Medical Sciences (Beijing, China). Prior to surgery, rats were acclimated for 1 week and housed in a temperature-controlled environment with a 12 h light/dark cycle and *ad libitum* access to food and water.

### Cerebral ischemia-reperfusion model

The MCAO surgery was conducted as previously described with several modifications [28, 29]. All rats were anesthetized with 1% pentobarbital sodium (50 mg/kg) by intraperitoneal injection followed by inserting an intraluminal suture from the external carotid artery stump into the internal carotid artery. And 2 h later, the suture was withdrawn to recover blood circulation. The rats of sham group underwent the same surgery without ligating the arteries. Subsequently, animals were randomly assigned into six groups (*n* = 10): sham, model, RD-1-L (5 mg/kg), RD-1-M (10 mg/kg), RD-1-H (20 mg/kg) and Ginaton (50 mg/kg) groups. RD-1 with a purity of more than 98% was synthesized and structurally identified as previously reported, [10] which was suspended in 0.5% (w/v) sodium carboxymethyl cellulose (CMC-Na) solution. 24 h after the start of reperfusion, vehicle or RD-1 were administered intragastrically and Ginaton was injected intraperitoneally once a day for 14 days until the animals were sacrificed. Sham and model groups received an equivalent volume of CMC-Na solution.

### Neurological function assessment

For all animals, the neurological function assessment was performed in 1, 3, 7 and 14 days after MCAO by an investigator who was blinded to experimental design. Neurological deficits were evaluated as described previously, [30] by a set of modified NSS tests, involving a series of motor, sensory, reflex, and balance measurements [31]. In the test, neurological function was graded from 0 to 18 (normal score, 0; maximal deficit score, 18).

### Adhesive-removal test

The adhesive-removal test was carried out in 14 days after MCAO as reported previously with several modifications [30]. In brief, two small pieces of adhesive-backed paper dots (of equal size, 100 mm^2^) were used as bilateral tactile stimuli occupying the distal-radial region on the wrist of each forelimb, the rats were then returned into the hyaline cage for observation. The time to remove each stimulus from forelimbs was recorded. All rats were familiarized with the test environment prior to testing. The maximum time allowed for the testing was 2 min.

### Infarct volume

In 14 days after MCAO, rats were anesthetized with 1% pentobarbital sodium (50 mg/kg) intraperitoneally and perfused firstly with 0.1 mol/L phosphate buffer saline (PBS) then 4% paraformaldehyde (pH 7.4) through the left ventricle. The brains were harvested and post fixed in 4% paraformaldehyde solution at 4°C, followed by achievement of equilibrium with 0.1 mol/L PBS containing 15%, 20% and 30% sucrose at 4°C, respectively. Each brain was coronally sectioned (2-mm thick) into five segments, from frontal to occipital pole. To measure the infarct volume coronal sections (10-μm thick) from each segment were then prepared for hematoxylin-eosin (HE) staining using a cryostat [32]. After staining, the sections were photographed using a digital camera and analyzed by Image-Pro Plus 6.0. The infarct volumes of the lesion structures were expressed as a percentage of the volume of the structures in the control hemispheres by using the formula of [(V_C_-V_L_)/V_C_] × 100%, where V_C_ is the volume of control hemisphere and V_L_ is the volume of non-infarcted tissue in the lesion hemisphere [33]. The total infarct volume of each brain was calculated as the sum of the infarct volumes of the five brain slides.

### Primary cortical neurons culture

Newborn Sprague-Dawley rats within 24 h were obtained from Weitonglihua Experimental Animal Centre (Beijing, China). Briefly, rats were disinfected in 75% ethanol for 10 s followed by brain harvest. The cortex of brain tissue was dissected and cut up into small tissue blocks. The precipitates were digested with 0.05% tyrisin for 10 min at 37°C. Finally, cells were filtered and suspended in DMEM medium containing 10% fetal bovine serum, and seeded on 8-well LabTek coverglass chambers or 96-well and 6-well plates pre-coated with 50 mg/ml poly-D-lysine and maintained at 37°C in a 5% CO_2_ environment. 6 h after plating, all medium was exchanged to serum-free Neurobasal medium with 2% B27 supplement. Subsequently half of the medium was renewed every 3 or 4 days. Most of experiments were performed between days *in vitro* (DIV) 12-14.

### OGD/R and measurement of cellular viability

To mimic the ischemia-reperfusion injury *in vitro*, OGD/R model was performed by using Na_2_S_2_O_4_ based on the method previously described with some modifications [34]. Briefly, in the OGD/R group, PCNs were cultured in glucose-free Neurobasal medium supplemented with 5 mmol/L Na_2_S_2_O_4_ for 1 h followed by replacement with normal medium. In the RD-1 treatment group, cells were exposed to 0, 0.1, 1, 5 and 10 μmol/L RD-1 while reperfusion. Control cultures were treated in a similar way except the hypoxic challenge. After 48 h treatment, cellular viability was measured by CCK-8 assay. In brief, 10 μL of CCK-8 was added to each well and then cells were incubated for 2 h at 37°C. The optical density at 450 nm was measured using a microplate reader.

### Measurement of mitochondrial ROS and MMP

ROS generation was measured by fluorescent probe 2’,7’-dichlorofluorescein diacetate (DCFH-DA). After 12 h exposure to 5 μmol/L RD-1, cells were washed twice with PBS, and then incubated with 10 μmol/L DCFH-DA for 20 min at 37°C. The extracellular DCFH-DA was removed by washing three times with Neurobasal medium free of B27 and then photographed using Olympus IX71 inverted fluorescent microscope. The relative fluorescence intensity was quantified using IPP 6.0 software.

MMP was measured by cationic dye JC-1 which accumulates in mitochondria in a potential-dependent manner. In normal mitochondria, JC-1 polymers produce strong red fluorescence in mitochondria matrix. In unhealthy mitochondria, JC-1 accrues in the cytosol in the form of monomer, producing green fluorescence. After 12 h treatment with 5 μmol/L RD-1, cells were washed twice with PBS, and then incubated with 10 mg/mL JC-1 for 15-20 min at 37°C. Then the cells were washed three times before they were suspended in incubation buffer. The images were captured using Olympus IX71 inverted fluorescent microscope and counted blindly. Mitochondrial depolarization degree was expressed as the ratio of red/green neurons numbers.

### Neuron transfection and live cell imaging of mitochondrial movement

Neurons plated in 8-well Lab-Tek chamber slides at different DIV were used and transfected 2 days before the imaging experiments, using Lipofectamine 2000 transfection reagent according to the manufacturer's instructions. After transfection, neurons were subjected to OGD/R and after 12 h treatment of RD-1, fluorescent time-lapse recordings were performed on the Live Cell Station (Olympus, Japan) [35]. For imaging of mitochondrial transport, we typically recorded neurons at a sampling rate of one frame every 5 s for 5 min, with the CCD exposure at 50 ms and 22 binning. A moving mitochondrion was defined as one that moved more than twice its length over the 5-min period. The velocity of mitochondrial transport was assessed using Velocity Demo software.

### Vesicle release assay

After 12 h treatment in the absence and presence of 5 μmol/L RD-1, all neurons were loaded with FM1-43 (10 mmol/L, Invitrogen) as previously described for 2 min. Coverslips were then washed with low K+ buffer for 5 min to remove excess dye [36, 37]. Images were acquired with a Cascade II EMCCD camera on an Olympus X61 inverted microscope. Images were collected with 200 ms exposures at 1 s interval during dye destaining. A baseline was collected for 10 images before addition of depolarizing buffer to destain the boutons. Dye-labeled boutons were selected as regions of interest in MetaMorph (Improvision), and fluorescence intensity was analyzed using IPP 6.0 software.

### Western blotting

For the animal samples, the total proteins were extracted from the ischemic cortex in 14 days after MCAO. For the cell samples, PCNs were dissected and homogenized in lyses buffer at 12 h after OGD/R. Western blots were performed as previously reported [29]. Equal amount of samples from each group were separated by SDS polyacrylamide gel electrophoresis and transferred onto PVDF membrane. The membranes were blocked in 5% skim milk for 1 h at room temperature and then incubated with primary antibody (KIF1B, Miro2, SNAP25, Complexin-1/2) overnight at 4°C, followed by secondary antibody conjugated to horseradish peroxidase for 1 h. Immunoblot was visualized with enhanced chemiluminescence and analyzed with GelPro software.

### Statistical analysis

All data were analyzed by One-way Analysis of Variance using SPSS 17.0 software. Multiple comparison post-hoc tests between groups were performed with Least-Significant Difference test or Dunnett's post-hoc test depending on homogeneity of variance test. Data were presented as mean ± SD, and differences between groups were considered significant at *P* < 0.05.

## Abbreviations

RD-1: 5-(4-hydroxy-3-dimethoxybenzylidene)-2-thioxo-4-thiazolidinone
MMP: mitochondrial membrane potential
ROS: radical oxidative species
MCAO: middle cerebral artery occlusion
PCNs: primary cortical neurons
OGD/R: oxygen-glucose deprivation and reoxygenation
NSS: Neurological Severity Scores
CMC-Na: sodium carboxymethyl cellulose
PBS: phosphate buffer saline
HE: hematoxylin-eosin
DIV: days *in vitro*
DCFH-DA: 2’,7’-dichlorofluorescein diacetate
